# The *fester* locus in *Botryllus schlosseri* experiences selection

**DOI:** 10.1186/1471-2148-12-249

**Published:** 2012-12-22

**Authors:** Marie L Nydam, Anthony W De Tomaso

**Affiliations:** 1 Division of Science and Mathematics, Centre College, 600 W. Walnut Street, Danville, KY, 40422, USA; 2 Department of Molecular, Cellular, and Developmental Biology, University of California, Santa Barbara, Santa Barbara, CA, 93106, USA

**Keywords:** Allorecognition, Selection, *Fester*, *Botryllus schlosseri*

## Abstract

**Background:**

Allorecognition, the ability of an organism to distinguish self from non-self, occurs throughout the entire tree of life. Despite the prevalence and importance of allorecognition systems, the genetic basis of allorecognition has rarely been characterized outside the well-known MHC (Major Histocompatibility Complex) in vertebrates and SI (Self-Incompatibility) in plants. Where loci have been identified, their evolutionary history is an open question. We have previously identified the genes involved in self/non-self recognition in the colonial ascidian *Botryllus schlosseri*, and we can now begin to investigate their evolution. In *B. schlosseri,* colonies sharing 1 or more alleles of a gene called FuHC (Fusion Histocompatibility) will fuse. Protein products of a locus called *fester*, located ~300 kb from FuHC, have been shown to play multiple roles in the histocompatibility reaction, as activating and/or inhibitory receptors. We test whether the proteins encoded by this locus are evolving neutrally or are experiencing balancing, directional, or purifying selection.

**Results:**

Nearly all of the variation in the *fester* locus resides within populations. The 13 housekeeping genes (12 nuclear genes and mitochondrial cytochrome oxidase I) have substantially more structure among populations within groups and among groups than *fester*. All polymorphism statistics (Tajima's D, Fu and Li's D* and F*) are significantly negative for the East Coast A-type alleles, and Fu and Li's F* statistic is significantly negative for the West Coast A-type alleles. These results are likely due to selection rather than demography, given that 10 of the housekeeping loci have no populations with significant values for any of the polymorphism statistics. The majority of codons in the *fester* proteins have ω values < 1, but 15–27 codons have > 95% posterior probability of ω values > 1.

**Conclusion:**

*Fester* proteins are evolving non-neutrally. The polymorphism statistics are consistent with either purifying selection or directional selection. The ω statistics show that the majority of the protein is experiencing purifying selection (ω < 1), but that 15–27 codons are undergoing either balancing or directional selection: ω > 1 is compatible with either scenario. The distribution of variation within and among populations points towards balancing selection and away from directional selection. While these data do not provide unambiguous support for a specific type of selection, they contribute to our evolutionary understanding of a critical biological process by determining the forces that affect loci involved in allorecognition.

## Background

Allorecognition is the ability of an organism to differentiate self or close relatives from unrelated individuals. Examples of allorecognition include the self-incompatibility (SI) systems in plants, vertebrate immune response to foreign antigens mediated by MHC loci, and fusion/rejection, where two genetically independent individuals physically join to become a single individual or reject each other. Effective allorecognition systems are critical to the survival of organisms: the SI loci prevent inbreeding depression, T-lymphocytes educated by MHC molecules protect vertebrates against pathogens, and fusing to a closely related individual can provide competitive and reproductive advantages where space is limited and reproductive output is based on the size of the organism [1]. Allorecognition occurs across the tree of life [1], in anemones [2], angiosperms [3], ascidians [4-6], bacteria [7], bryozoans [8], cellular slime molds [9], corals [10], fungi [11], hydroids [12], gymnosperms [13,14], plasmodial slime molds [15], red algae [16], sponges [17], and vertebrates [18].

Despite the prevalence and importance of allorecognition systems, the genetic basis of allorecognition has rarely been characterized outside the well-known MHC in vertebrates and SI in plants. The genes responsible for allorecognition have recently been identified in a handful of systems: a bacterium [7], a colonial ascidian [19], a cellular slime mold [20], fungi [11], a hydroid [21], and a solitary ascidian [6]. Only in the ascidian systems have we identified putative receptor-ligand pairs [22]; ligands bind to receptors on the cell surface.

In the colonial ascidian *Botryllus schlosseri,* allorecognition occurs when terminal projections of an extracorporeal vasculature, called ampullae, come into contact between juxtaposed colonies. If colonies are compatible, the ampullae will fuse, forming a parabiosis between the two colonies. If they are incompatible, the ampullae will undergo a rejection reaction which prevents vascular fusion. The polymorphisms of a gene called FuHC (Fusion/HistoCompatibility) determine 100% of histocompatibility outcomes between interacting colonies: fusion occurs if the colonies share 1 or more FuHC alleles [19, Nydam et al., unpublished data].

Another polymorphic locus, called *fester,* is encoded ~300 Kb from the FuHC; FuHC and *fester* are tightly linked [22]. *Fester* appears to encode a cell-surface receptor involved in multiple aspects of histocompatibility in *B. schlosseri*. From a genetic standpoint, *fester* displays a characteristic reminiscent of all immune genes – diversity.

*Fester* achieves diversity through several mechanisms. First, the locus is highly polymorphic, and encodes over 60 protein alleles, although these polymorphisms do not contribute to histocompatibility outcomes [22]. Because *fester* is likely a receptor of the ligand FuHC [22], these polymorphisms can tell us how *fester* and FuHC interact at the molecular level to determine histocompatibility outcomes. Specifically, regions of high polymorphism in *fester* could indicate domains that bind with FuHC, and vice versa.

**Figure 1:**
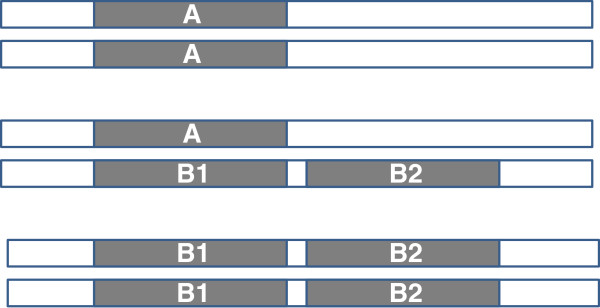
**Cartoon of the *fester *genomic structure.** Top panel: A/A homozygote, Middle Panel: A/B1/B2 heterozygote, Bottom Panel: B1/B2/B1/B2 heterozygote. The C haplotype is not included because its composition and location relative to the A and B haplotypes is unknown.

Second, a preliminary phylogenetic analysis of *fester* coding sequences split them into 4 groups: A, B1, B2, and C [22]. The A haplotype has a single copy of *fester*, while the B haplotype encodes 2 linked duplicates (B1 and B2). At present, the composition of the C haplotype is unknown. There are no other *fester* genes anywhere else, based on our crossing data [22]. We will use the term haplotype to refer to the A, B1/B2 and C haplotypes in this article. Figure 1 provides a visual representation of these haplotypes.

Finally, the *fester* locus is diversified in each colony via alternative splicing [22]. The *fester* locus is encoded in 11 exons, 6 of which can be alternatively spliced in all combinations, making a total of 64 potential splice variants. Each colony examined expresses a full-length *fester* mRNA, 3 common alternative splice variants, as well as a unique repertoire of 8–24 different alternative splice variants; Exons 6 and 7 are very commonly spliced out [22]. Exons 1–7 correspond to the extracellular domain of the protein, and Exons 8–10 to 3 predicted transmembrane domains [22]. In the present data set, the PCR primers always amplified all 11 exons. However, the full-length cDNA was rarely incorporated into the bacterial vector; the longest amplicons recovered from the cloning process were almost always missing Exons 6 and 7.

Functionally, 2 experiments support a role of the *fester* protein in histocompatibility. A monoclonal antibody (mAB) experiment, whereby the histocompatibility reaction is interrupted by a mAB that binds to and activates the *fester* protein, resulted in a rejection being converted to a fusion. This conversion only occurred in pairings involving *fester* genotypes that expressed the *fester* allele that binds to the mAB [22]. *Fester* could therefore be a receptor which binds to FuHC, blocking an ongoing rejection reaction and initiating the fusion event [22]. In contrast, a siRNA experiment, in which the expression of the *fester* protein is blocked, turned both fusion and rejection phenotypes into no response phenotypes, and ampullae were inert [22]. This result suggests that *fester* also plays a role in initiating the rejection reaction. Given this dual role, if *fester* is not expressed, no histocompatibility reaction occurs: a rejection reaction cannot occur because it is not initiated, and a fusion cannot occur because no receptors can detect the FuHC.

We know very little about the evolution of allorecognition loci outside MHC and SI, but we have several reasons to hypothesize that loci like *fester* may be evolving non-neutrally. First, abundant evidence exists for selection acting on both ligands and receptors directly involved in MHC (reviewed in [23,24]) and SI (reviewed in [25]). Second, *fester* is highly polymorphic [22]; a neutral model of evolution is unlikely to explain allelic diversity found in *fester* and other allorecognition loci [26].

We will use three approaches to infer whether *fester* is experiencing selection or genetic drift: distribution of polymorphism within and among populations (AMOVA and F_ST_ calculations), polymorphism statistics, and ω statistics. AMOVA and F_ST_ values for *fester* alleles will be compared to housekeeping genes. If the *fester* alleles are outliers with respect to the housekeeping loci, this will be taken as evidence for selection. Polymorphism statistics (D, D*, F*) look for evidence of selection using a genealogical framework; values significantly different from zero are evidence for selection. The ω statistic calculates the posterior probability that particular codons are experiencing selection.

If selection is acting on *fester,* we will examine the support for three types of selection: balancing, directional, and purifying. Using AMOVA and F_ST_ statistics, a low amount of polymorphism within populations compared to housekeeping genes is consistent with directional selection; the opposite pattern is consistent with balancing selection [27]. Polymorphism statistics (D, D*, F*) are less than zero when purifying or directional selection is operating, and greater than zero when balancing selection is operating [28,29]. An ω value greater than 1 supports directional or balancing selection, and less than 1 supports purifying selection [30]. In the few cases where allorecognition loci have been studied in an evolutionary framework, balancing selection is more prevalent than purifying or directional selection [25]. If we find that *fester* alleles evolve under selection, we therefore expect to find evidence for balancing selection.

## Results

### Sampling

Colonies were collected from floating docks in each of 6 populations in 2009 and 2010: Falmouth, MA, Quissett, MA, Sandwich, MA, Monterey, CA, Santa Barbara, CA and Seattle, WA. Falmouth, MA and Quissett, MA are 3 miles apart, on Vineyard Sound and Buzzards Bay, respectively. Sandwich, MA is 25 miles from the Falmouth/Quissett area, on Cape Cod Bay. Santa Barbara, CA is 237 miles south of Monterey, CA and 1,113 miles south of Seattle, WA.

### Relationships among *fester* haplotypes

**Figure 2:**
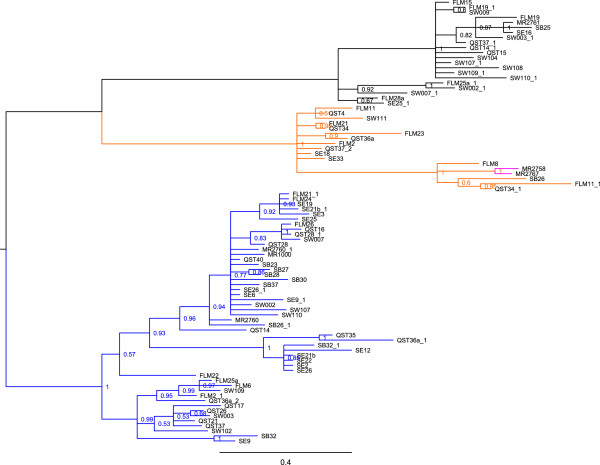
**Unrooted 50% majority-rule Bayesian consensus tree of *fester *A, B1, B2 and C allele types.** The values are posterior probability values. Blue = A-type alleles, Orange = B1-type alleles, Purple = B2-type alleles, Black = C-type alleles. FLM = Falmouth MA, MR = Monterey CA, QST = Quissett MA, SW = Sandwich MA, SB = Santa Barbara CA, SE = Seattle WA.

We constructed phylogenetic trees to evaluate the evolutionary relationships between the *fester* haplotypes. ML and Bayesian methods show the A-type alleles, the B1/B2-type alleles and the C-type alleles to be monophyletic groups (Figure 2, Additional file 1). In all analyses, the 2 B2-type alleles group together, but B1 is paraphyletic with respect to B2. The B2 clade has strong support (Bayesian posterior probability = 1.0, ML bootstrap value = 0.93).

### Comparison of variation among *fester* allele types

We compared variation and diversity among *fester* allele types to inform our understanding of the evolution of these allele types. Significantly more variation exists in the A clade than in either B1/B2 or C. D_xy_/D_a_ values quantify this disparity: A clade vs. B1/B2 clade = 0.061/0.050, A clade vs. C clade = 0.061/0.051, B1/B2 clade vs. C clade = 0.047/0.032. Because D_a_ corrects for within-allele type variation, the higher variation in the A-type alleles is not an artifact of more colonies having A-type alleles than B1/B2 or C-type alleles.

When A-type alleles are compared between East Coast and West Coast, several measures of diversity (π based on all sites, π based on synonymous sites, Watterson's Θ, and haplotype diversity) are larger for the East Coast group than for the West Coast group (Table 1). We sampled 22 colonies with A-type alleles from the East Coast, and 18 colonies with A-type alleles from the West Coast.

**Table 1:** π, θ-w, # haplotypes, haplotype diversity

**Fester A-type alleles**						
Population	π (All Sites)	π (Synonymous)	π (Nonsynonymous)	Θ-w (per site)	# Haplotypes	Haplotype diversity
East Coast	0.020	0.020	0.010	0.030	17	0.96
West Coast	0.010	0.010	0.010	0.010	13	0.90
**Fester B1-type alleles**						
Population	π (All Sites)	π (Synonymous)	π (Nonsynonymous)	Θ-w (per site)	# Haplotypes	Haplotype diversity
East Coast	0.010	0.030	0.010	0.010	8	0.95
**Fester C-type alleles**						
Population	π (All Sites)	π (Synonymous)	π (Nonsynonymous)	Θ-w (per site)	# Haplotypes	Haplotype diversity
East Coast	0.010	0.030	0.010	0.020	13	0.93
**40S_3A**						
Population	π (All Sites)	π (Synonymous)	π (Nonsynonymous)	Θ-w (per site)	# Haplotypes	Haplotype diversity
Falmouth, MA	0.001	0.014	0.000	0.002	6	0.533
Quissett, MA	0.001	0.004	0.000	0.001	3	0.600
Sandwich, MA	0.002	0.011	0.000	0.002	3	0.833
Monterey, CA	NA	NA	NA	NA	1	0.000
Santa Barbara, CA	0.006	0.025	0.000	0.006	2	0.500
Seattle, WA	0.009	0.040	0.000	0.007	3	0.800
All populations	0.003	0.014	0.000	0.005	9	0.658
**60S_L6**						
Population	π (All Sites)	π (Synonymous)	π (Nonsynonymous)	Θ-w (per site)	# Haplotypes	Haplotype diversity
Falmouth, MA	0.002	0.006	0.001	0.003	5	0.455
Quissett, MA	0.001	0.003	0.000	0.002	5	0.433
Sandwich, MA	0.002	0.005	0.000	0.002	5	0.547
Monterey, CA	0.008	0.015	0.006	0.008	3	0.511
Santa Barbara, CA	0.002	0.004	0.002	0.004	8	0.699
Seattle, WA	0.002	0.007	0.001	0.003	8	0.678
All populations	0.003	0.008	0.002	0.007	18	0.711
**60S_L8**						
Population	π (All Sites)	π (Synonymous)	π (Nonsynonymous)	Θ-w (per site)	# Haplotypes	Haplotype diversity
Falmouth, MA	0.001	0.003	0.001	0.002	5	0.524
Quissett, MA	0.002	0.004	0.001	0.002	5	0.599
Sandwich, MA	0.002	0.003	0.001	0.002	3	0.833
Monterey, CA	0.001	0.003	0.000	0.001	2	0.500
Santa Barbara, CA	NA	NA	NA	NA	NA	NA
Seattle, WA	0.001	0.002	0.001	0.001	3	0.506
All populations	0.002	0.003	0.001	0.001	5	0.593
**60S_L10**						
Population	π (All Sites)	π (Synonymous)	π (Nonsynonymous)	Θ-w (per site)	# Haplotypes	Haplotype diversity
Falmouth, MA	0.005	0.020	0.000	0.007	7	0.393
Quissett, MA	0.007	0.032	0.000	0.007	6	0.693
Sandwich, MA	0.003	0.014	0.000	0.004	2	0.333
Monterey, CA	0.009	0.039	0.000	0.009	6	0.929
Santa Barbara, CA	0.010	0.041	0.000	0.009	7	0.911
Seattle, WA	0.008	0.035	0.000	0.005	10	0.776
All populations	0.008	0.036	0.000	0.007	18	0.672
**60S_L13**						
Population	π (All Sites)	π (Synonymous)	π (Nonsynonymous)	Θ-w (per site)	# Haplotypes	Haplotype diversity
Falmouth, MA	0.007	0.018	0.004	0.005	13	0.833
Quissett, MA	0.008	0.019	0.004	0.006	7	0.655
Sandwich, MA	0.006	0.016	0.004	0.007	2	0.500
Monterey, CA	0.003	0.009	0.001	0.002	3	0.644
Santa Barbara, CA	0.007	0.021	0.003	0.005	4	0.714
Seattle, WA	0.008	0.026	0.003	0.006	16	0.903
All populations	0.010	0.028	0.004	0.006	19	0.833
**71kda**						
Population	π (All Sites)	π (Synonymous)	π (Nonsynonymous)	Θ-w (per site)	# Haplotypes	Haplotype diversity
Falmouth, MA	0.004	0.014	0.000	0.005	10	0.804
Quissett, MA	0.004	0.014	0.001	0.005	9	0.735
Sandwich, MA	0.006	0.023	0.001	0.006	13	0.896
Monterey, CA	0.009	0.037	0.000	0.007	5	0.867
Santa Barbara, CA	0.008	0.032	0.000	0.006	19	0.943
Seattle, WA	0.004	0.014	0.001	0.004	7	0.909
All populations	0.008	0.029	0.001	0.007	47	0.906
**actin**						
Population	π (All Sites)	π (Synonymous)	π (Nonsynonymous)	Θ-w (per site)	# Haplotypes	Haplotype diversity
Falmouth, MA	0.004	0.017	0.000	0.005	12	0.879
Quissett, MA	0.006	0.025	0.000	0.005	14	0.931
Sandwich, MA	0.005	0.021	0.000	0.006	12	0.918
Monterey, CA	0.004	0.015	0.000	0.004	4	0.652
Santa Barbara, CA	0.005	0.022	0.000	0.005	18	0.954
Seattle, WA	0.005	0.020	0.000	0.004	6	0.818
All populations	0.005	0.022	0.000	0.007	44	0.924
**ADP/ATP translocase**						
Population	π (All Sites)	π (Synonymous)	π (Nonsynonymous)	Θ-w (per site)	# Haplotypes	Haplotype diversity
Falmouth, MA	0.008	0.029	0.001	0.007	13	0.863
Quissett, MA	0.014	0.052	0.002	0.014	3	0.833
Sandwich, MA	0.005	0.020	0.000	0.005	3	0.833
Monterey, CA	0.020	0.082	0.000	0.020	2	1.000
Santa Barbara, CA	NA	NA	NA	NA	NA	NA
Seattle, WA	0.012	0.046	0.001	0.012	4	1.000
All populations	0.009	0.034	0.001	0.007	18	0.915
**Hsp90**						
Population	π (All Sites)	π (Synonymous)	π (Nonsynonymous)	Θ-w (per site)	# Haplotypes	Haplotype diversity
Falmouth, MA	0.015	0.065	0.001	0.020	13	0.788
Quissett, MA	0.010	0.044	0.001	0.016	13	0.812
Sandwich, MA	0.018	0.079	0.001	0.012	7	0.911
Monterey, CA	0.002	0.008	0.000	0.002	8	0.909
Santa Barbara, CA	0.002	0.008	0.000	0.002	9	0.815
Seattle, WA	0.014	0.063	0.001	0.013	3	0.464
All populations	0.010	0.045	0.001	0.014	23	0.685
**ma2_actin**						
Population	π (All Sites)	π (Synonymous)	π (Nonsynonymous)	Θ-w (per site)	# Haplotypes	Haplotype diversity
Falmouth, MA	0.003	0.013	0.000	0.004	9	0.831
Quissett, MA	0.004	0.016	0.000	0.004	8	0.857
Sandwich, MA	0.003	0.011	0.000	0.004	7	0.768
Monterey, CA	0.004	0.017	0.000	0.006	3	0.385
Santa Barbara, CA	0.004	0.016	0.000	0.004	4	0.458
Seattle, WA	0.003	0.016	0.000	0.003	4	0.542
All populations	0.005	0.019	0.000	0.006	13	0.777
**mtCOI**						
Population	π (All Sites)	π (Synonymous)	π (Nonsynonymous)	Θ-w (per site)	# Haplotypes	Haplotype diversity
Falmouth, MA	0.009	0.031	0.002	0.016	4	0.491
Quissett, MA	0.007	0.035	0.002	0.010	5	0.405
Sandwich, MA	0.026	0.094	0.004	0.158	3	0.833
Monterey, CA	0.002	0.007	0.000	0.003	2	0.400
Santa Barbara, CA	0.004	0.015	0.002	0.010	6	0.468
Seattle, WA	0.025	0.105	0.002	0.018	4	0.711
All populations	0.021	0.093	0.002	0.013	11	0.725
**vasa**						
Population	π (All Sites)	π (Synonymous)	π (Nonsynonymous)	Θ-w (per site)	# Haplotypes	Haplotype diversity
Falmouth, MA	0.004	0.011	0.001	0.004	6	0.733
Quissett, MA	0.020	0.080	0.002	0.020	7	0.800
Sandwich, MA	0.023	0.095	0.000	0.020	10	0.970
Monterey, CA	0.012	0.047	0.001	0.010	5	0.857
Santa Barbara, CA	0.025	0.097	0.003	0.020	8	0.859
Seattle, WA	0.013	0.055	0.000	0.011	5	0.861
All populations	0.022	0.088	0.002	0.026	23	0.750
**Vigilin**						
Population	π (All Sites)	π (Synonymous)	π (Nonsynonymous)	Θ-w (per site)	# Haplotypes	Haplotype diversity
Falmouth, MA	0.011	0.037	0.004	0.017	9	0.934
Quissett, MA	0.023	0.080	0.007	0.017	15	0.980
Sandwich, MA	0.019	0.065	0.007	0.016	11	0.958
Monterey, CA	0.018	0.069	0.004	0.018	5	0.893
Santa Barbara, CA	0.018	0.070	0.004	0.016	23	0.938
Seattle, WA	0.018	0.064	0.004	0.017	5	0.822
All populations	0.024	0.088	0.006	0.016	55	0.971

### Recombination

All populations (A-type East and West Coasts, B1-type and C-type East Coast) experience intragenic recombination. 2 and 1 minimum number of recombination events (R_m_) are found in the A-type East and West Coast groups, respectively. For A-type East Coast, recombination is detected between sites 279,300 (in Exon 3) and 338,731 (between Exons 4 and 8). For A-type West Coast, recombination is detected between sites 447,701 (between Exons 4 and 8). Significant negative correlations between physical distance and all 3 measures of LD for East Coast A-type alleles, and for 1 measure of LD for West Coast A-type alleles (p = 0.001 in all significant negative correlations). The East Coast B1-type and C-type alleles have 0 minimum recombination events, but significant negative correlations between physical distance and all 3 measures of LD are found in both groups (p = 0.001 in all significant negative correlations).

### Selection inference: distribution of polymorphism within and among populations

#### *Fester*

Analyzing the distribution of *fester* polymorphism within and among populations and comparing these distributions to housekeeping genes allows us to make inferences about whether selection is occurring at *fester* alleles. AMOVA, fixation indices (F_ct_, F_sc_ and F_st_), and pairwise F_st_ values for all allele types are shown in Additional file 2. The two groups are all East Coast populations taken together, and all West Coast populations taken together. For the A-type alleles, 96% of the variation is found within populations and there is no significant differentiation among groups, among populations within groups, or among populations among groups. Only 1/15 pairwise F_st_ values is significant (Quissett, MA vs. Santa Barbara, CA). Qualitative results are identical for the B1-type and C-type alleles: 100% of the variation is found within (as opposed to among) populations, neither global F_st_ nor pairwise F_st_ values are significant. Clearly, *fester* variation is found exclusively within populations.

The allele types show some geographic signature. The A-type alleles, by far the most common (present in 71% of colonies sequenced), are found in similar numbers in East Coast and West Coast populations (27 East Coast alleles, 24 West Coast alleles), but the B1-type and C-type allele types are rarely found on the West Coast (2/13 B1-type alleles and 4/21 C-type alleles). Only 2 B2-type alleles are present in our samples, and although they are both found in the Monterey population, we cannot conclude anything about the geographic structuring of this allele type with so few samples.

#### Housekeeping genes

**Figure 3:**
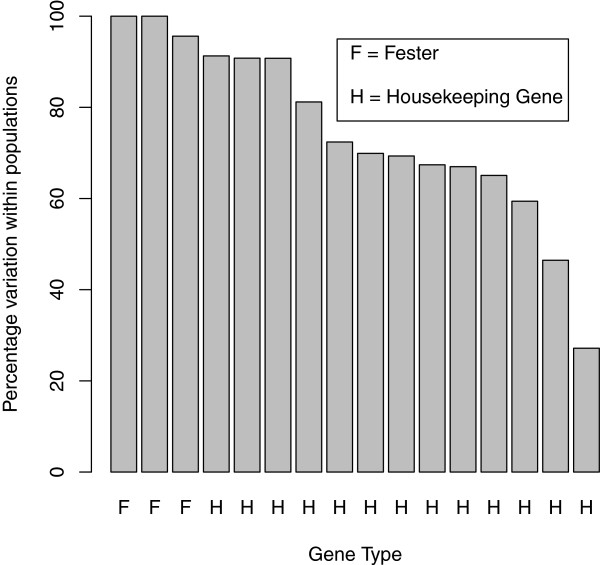
**Comparison of percentage of variation found within populations between *fester *and housekeeping genes.***Fester* types are labeled "F", and housekeeping genes are labeled "H". The three *fester* values are A-type, B1-type and C-type. These numbers were derived from AMOVA (analyses of molecular variance), which are presented in their entirety in Additional file 2. The bars from left to right correspond to the following genes: *fester* B1-type, *fester* C-type, *fester* A-type, cytoplasmic actin 2, 60S ribosomal protein L10, 60S ribosomal protein L13, mitochondrial COI, 60S ribosomal protein L8, 60S ribosomal protein L6, 40S ribosomal protein 3A, ADP/ATP translocase 3, adult-type muscle actin 2, vigilin, heat shock cognate 71 kda protein, heat shock protein HSP-90 beta, and vasa.

The housekeeping genes are as follows: mitochondrial COI, 40S ribosomal protein 3A, 60S ribosomal protein L6, 60S ribosomal protein L8, 60S ribosomal protein L10, 60S ribosomal protein L13, adult-type muscle actin 2, heat shock cognate 71kda protein, cytoplasmic actin 2, ADP/ATP translocase 3, heat shock protein HSP-90 beta, vasa, and vigilin. The housekeeping genes show a pattern that contrasts with *fester*. All housekeeping loci have a substantially lower percentage of the variation within populations than *fester* (Figure 3). All loci have a substantially higher percentage of the variation among groups than *fester* although F_ct_ is not significant for any locus (Additional file 2). All loci have a substantially higher percentage of the variation among populations within groups than *fester*, and F_sc_ is significant for all loci but 1 (Additional file 2). Significant differentiation exists among populations among groups: overall F_st_ is significant for all loci_,_ and a majority of pairwise F_st_ values are significant.

### Tests of selection: polymorphism statistics

#### *Fester*

Table 1 shows θ and π for each allele type. For the A-type alleles, East Coast has higher levels of polymorphism than West Coast. B1-type and C-type East Coast groups have polymorphism values similar to those of the A-type West Coast group.

Table 2 displays Tajima's D values for A-type East and West Coasts, and B1-type/C-type East Coast. Coalescent simulations given θ and segregating sites show similar p-values (and always with the same qualitative result), so only results from the simulations given θ will be shown. A-type: D values are significantly negative for East Coast only. B1-type and C type East Coast D values are also negative, but are not statistically significant.

**Table 2:** Tajima's D, Fu and Li's D* and F* statistics for fester A, B1 and C allele types

**Allele type and Location**	**Tajima's D value**	Fu and Li's D* value	Fu and Li's F* value
A-type alleles East Coast	−1.31*	−1.96*	−1.96*
A-type alleles West Coast	−1.01	−0.85	−0.95*
B1-type alleles East Coast	−0.99	−0.27	−0.52
C-type alleles East Coast	−1.26	−1.51	−1.67

Asterisks represent D, D* or F* values with associated p values that are less than 0.05.

Fu and Li's D* and F* values are presented in Table 2. Coalescent simulations given θ and segregating sites show similar p-values (and always with the same qualitative result), so only results from the simulations given θ will be shown. Both A-type groups have significantly negative D* values, but only East Coast has significantly negative F* values. Just as in the Tajima's D analyses, B1-type and C-type East Coast D values are negative, but p values from the coalescent simulations are > 0.05 in all cases.

#### Housekeeping genes

Summary statistics, shown in Table 2, have consistently lower values for the housekeeping genes than for *fester*. Values for Tajima’s D, Fu and Li’s D* and F* can be found in Additional file 3. We see very few significant values for the housekeeping loci. For mtCOI, two populations are significant for all three statistics. For 60S ribosomal protein L10, one population is significant for all three statistics. For vasa, one population is significant for one statistic. None of the other housekeeping loci have populations with significant values for any of the three statistics.

We also noted that all D, D* and F* values across populations are negative for *fester*. For the housekeeping genes, only cytoplasmic actin 2 shows a pattern of consistent negative values across populations for the polymorphism statistics. 40S ribosomal protein 3A, 60S ribosomal protein L8, 60S ribosomal protein L10, ADP/ATP translocase, HSP 90 beta and mtCOI show no trend towards positive or negative values across populations for any of the three statistics. 60S ribosomal protein L6 and adult-type muscle actin 2 are negative across populations for Tajima’s D, but no pattern is seen in either D* or F*. Heat shock cognate 71kda protein and vasa have no pattern for Tajima’s D, but a majority of populations have positive values for D* and F*. 60S ribosomal protein L13 shows a pattern of positive values across populations for all three statistics.

**Figure 4:**
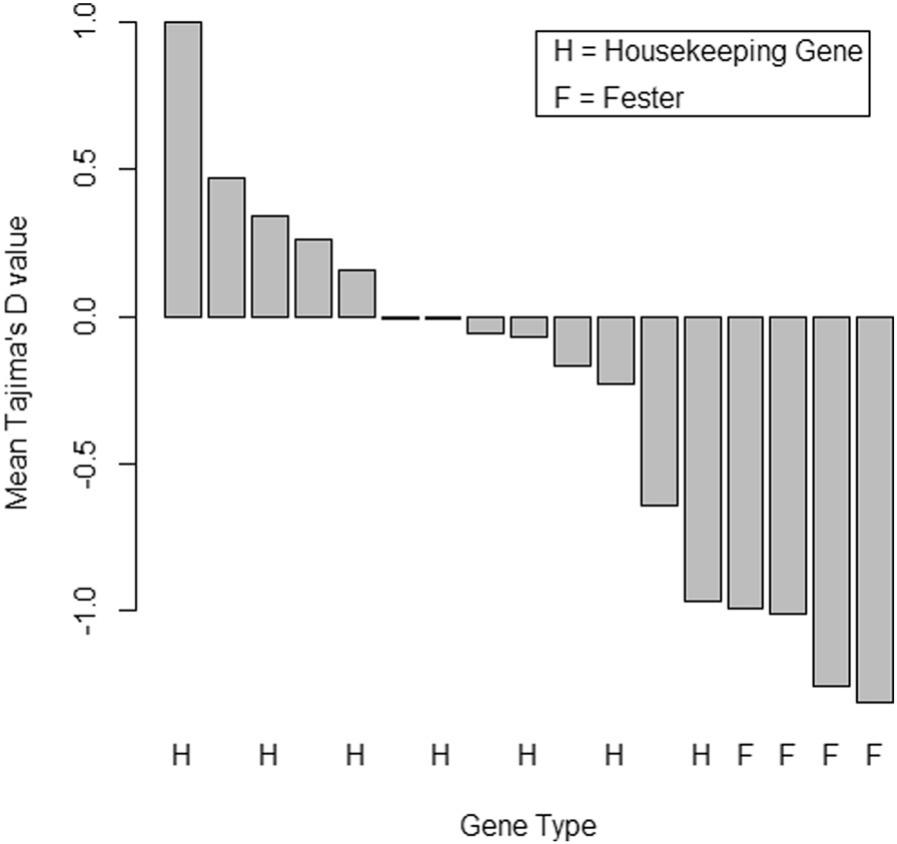
**Comparison of mean Tajima's D between *fester *and housekeeping genes. ***Fester* genes are labeled "F", and housekeeping genes are labeled "H". There are four *fester* values: A-type East Coast, A-type West Coast, B1-type East Coast, C-type East Coast. Mean Tajima's D values for housekeeping genes were obtained by averaging across all six populations (Falmouth, MA, Quissett, MA, Sandwich, MA, Monterey, CA, Santa Barbara, CA and Seattle, WA). The bars from left to right correspond to the following genes: 60S ribosomal protein L13, vasa, heat shock cognate 71kda protein, vigilin, 60S ribosomal protein L10, ADP/ATP translocase 3, heat shock protein HSP-90 beta, cytoplasmic actin 2, 60S ribosomal protein L8, mitochondrial COI, 40S ribosomal protein 3A, adult-type muscle actin 2, 60S ribosomal protein L6, *fester* B1-type East Coast*, fester* A-type West Coast, *fester* C-type East Coast, and *fester* A-type East Coast.

**Figure 5:**
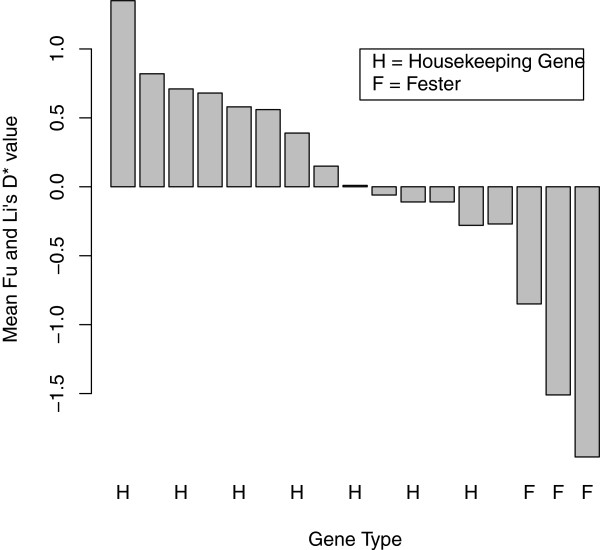
Comparison of mean Fu and Li's D* between *fester *and housekeeping genes. *Fester* genes are labeled "F", and housekeeping genes are labeled "H". There are four *fester* values: A-type East Coast, A-type West Coast, B1-type East Coast, C-type East Coast. Mean Fu and Li's D* values for housekeeping genes were obtained by averaging across all six populations (Falmouth, MA, Quissett, MA, Sandwich, MA, Monterey, CA, Santa Barbara, CA and Seattle, WA). The bars from left to right correspond to the following genes: vasa, 60S ribosomal protein L13, vigilin, heat shock cognate 71kda protein, heat shock protein HSP-90 beta*,* mitochondrial COI, ADP/ATP translocase 3, cytoplasmic actin 2, 40S ribosomal protein 3A*,* adult-type muscle actin 2, 60S ribosomal protein L6, 60S ribosomal protein L10, 60S ribosomal protein L8, *fester* B1-type East Coast*, fester* A-type West Coast, *fester* C-type East Coast, and *fester* A-type East Coast.

**Figure 6:**
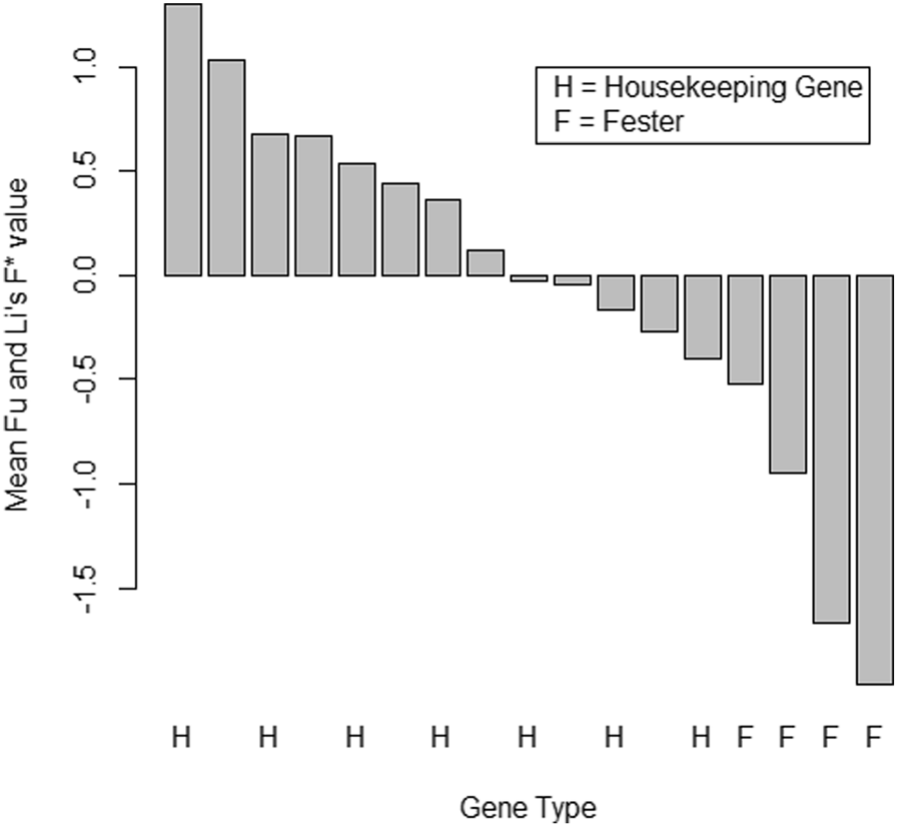
Comparison of mean Fu and Li's F* between *fester *and housekeeping genes. *Fester* genes are labeled "F", and housekeeping genes are labeled "H". There are four *fester* values: A-type East Coast, A-type West Coast, B1-type East Coast, C-type East Coast. Mean Fu and Li's F* values for housekeeping genes were obtained by averaging across all six populations (Falmouth, MA, Quissett, MA, Sandwich, MA, Monterey, CA, Santa Barbara, CA and Seattle, WA). The bars from left to right correspond to the following genes: vasa, 60S ribosomal protein L13, vigilin, heat shock cognate 71kda protein, mitochondrial COI, heat shock protein HSP-90 beta*,* ADP/ATP translocase 3, cytoplasmic actin 2, 40S ribosomal protein 3A*,* ribosomal protein L10, 60S ribosomal protein L8, adult-type muscle actin 2, 60S ribosomal protein L6, 60S *fester* B1-type East Coast*, fester* A-type West Coast, *fester* C-type East Coast, and *fester* A-type East Coast.

To further compare housekeeping genes and *fester*, we calculated mean Tajima's D, Fu and Li's D*, and Fu and Li's F* values (across all six populations) for housekeeping genes. We then compared these values to D, D* and F* for *fester* A-type East Coast, A-type West Coast, B1-type East Coast, and C-type West Coast (Figures 4, 5, 6). For all of the statistics, the *fester* values and the housekeeping gene values are non-overlapping. Specifically, all of the *fester* values for each statistic are lower than all of the housekeeping gene values. This clearly shows that *fester* does not experience the same evolutionary forces as the rest of the genome.

### Tests of selection: ω statistics

**Figure 7:**
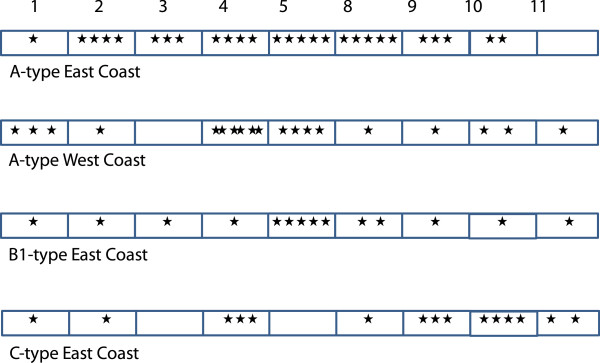
**Locations of the codons with greater than 95% probability of ω greater than 1 across the *fester *locus.** The numbers at the top of the figure refer to the exons sequenced. The analysis was done on four separate data sets - A-type East Coast alleles, A-type West Coast alleles, B1-type East Coast alleles and C-type East Coast alleles.

The locations and number of all codons with >95% posterior probability of directional/balancing selection are shown in Figure 7. Exons 1–7 are predicted to correspond to extracellular domains of the *fester* protein, Exons 8–10 to transmembrane domains. Codons with <95% posterior probability of selection are not considered to be under directional/balancing selection. The majority of codons in the *fester* proteins have ω values < 1, consistent with purifying selection.

A-type alleles East Coast. 27 codons have >95% posterior probability of directional/balancing selection (ω > 1). Exons 3, 4 and 8 have significantly higher ω values than the rest of the gene (Exon 3: W value = 5,616, p value = 0.020, Exon 4: W value = 8,037, p value = 0.007, Exon 8: W value = 6,981, p value = 0.013). A-type alleles West Coast. 19 codons have >95% posterior probability of directional/balancing selection (ω > 1). Exons 4, 5 and 8 have significantly higher ω values than the rest of the gene (Exon 4: W value = 7,983, p value = 0.0084, Exon 5: W value = 9,651, p value = 0.0105, Exon 8: W value = 7,478, p value = 0.0007). B1-type alleles East Coast. 17 codons have >95% posterior probability of directional/balancing selection (ω > 1). Exons 4, 5 and 8 have significantly higher ω values than the rest of the gene (Exon 4: W value = 8,032, p value – 0.014, Exon 5: W value = 11,062, p value = <0.001, Exon 8: W value = 7,230, p value = 0.0138). Exons 9–11 do not have significantly higher ω values than the rest of the gene. C-type alleles East Coast. 15 codons have >95% posterior probability of directional/balancing selection (ω > 1). Exons 4, 5 and 8 have significantly higher ω values than the rest of the gene (Exon 4: W value = 8,032, p value – 0.014, Exon 5: W value = 11,062, p value = <0.001, Exon 8: W value = 7,230, p value = 0.0138). Exons 9–11 do not have significantly higher ω values than the rest of the gene.

## Discussion

### Tests of selection

#### Selection is occurring at the *fester* locus

Two sets of hypotheses have generally been advanced for the maintenance of polymorphism at allorecognition loci – those invoking neutral processes, and those invoking selective processes (reviewed in [31]). All three analytical methods we employed (distribution of variation within and among populations, polymorphism statistics and ω statistics) provide evidence that selection is occurring at the *fester* locus.

Nearly all of the variation in the *fester* locus resides within populations, just as with FuHC [32]. The housekeeping genes have substantially more structure among populations within groups and among groups than *fester* (Figure 3). In addition, a majority of the pairwise F_st_ values are significant for the housekeeping genes, but only one is significant in *fester* A-type, and none in B1-type or C-type. The East Coast exhibits more differentiation between populations than the West Coast: 15 intra-East Coast population pairs show differentiation whereas 19 intra-West Coast population pairs do (Additional file 2). This variation in amount of differentiation may be due to the wider geographical sampling on the West Coast. However, any bias in AMOVA results due to higher differentiation on the West Coast than on the East Coast would affect both *fester* and housekeeping genes.

The population differentiation at the housekeeping loci confirms the significant genetic structure seen for neutral markers (microsatellites) in *B. schlosseri*[33,34]. These results are in sharp contrast to the lack of significant population differentiation at *fester* and FuHC.

The pattern seen here is consistent with balancing selection acting on *fester*. Loci experiencing balancing selection (which maintains variation) should have larger amounts of polymorphism within populations and smaller amounts among populations than neutral loci (assuming selection pressures are similar between populations), whereas the opposite pattern is expected for loci experiencing directional selection [27].

All polymorphism statistics (Tajima's D, Fu and Li's D* and F*) are significantly negative for the East Coast A-type alleles, and Fu and Li's F* statistic is significantly negative for the West Coast A-type alleles, consistent with either purifying selection or a recent selective sweep at this haplotype (directional selection). These results are likely due to selection rather than demography, given that 10 of the housekeeping loci have no populations that were significant for any of the polymorphism statistics. The remaining three loci only have 1–2 populations (out of six) that were significant for one or more of the statistics. In addition, the housekeeping loci do not show a consistent negative trend of polymorphism statistics across all populations, as *fester* does. Figures 4, 5, 6 provide additional conformation that values of polymorphism statistics are more negative for *fester* than for housekeeping genes.

Polymorphism statistics for B1-type and C-type alleles are always negative, but are not statistically different from zero. We cannot therefore reject the null hypothesis that the B1-type and C-type alleles are evolving neutrally, based on these statistics. However, *fester* B1-type and C-type are clearly on a different evolutionary trajectory than the rest of the *B. schlosseri* genome (Figures 4, 5, 6) and ω statistics provide evidence for selection on all 3 of the *fester*-allele types tested: A, B1 and C.

Exons 4,5 and 8 have statistically higher ω values than the rest of the gene for the A-type West Coast group, the B1-type group, and the C-type group. The A-type East Coast group also highlights Exon 3 as significant, but not Exon 5. No putative conserved domains were detected when Exons 3 and 4 were submitted as queries to the NCBI non-redundant protein sequences database using BLASTp. Exon 5 encodes a short consensus repeat (SCR, or sushi) domain often found in vertebrate complement receptors (part of the innate immune system) [22]. Exon 8 is a functional transmembrane domain that was co-localized with CD45 to the cellular membrane [22]. Splice variants missing Exons 3 and 5 are occasionally found, but Exons 4 and 8 are present in all variants sequenced thus far [22]. We will focus on these 4 exons in further studies of *fester*'s role in the allorecognition reaction, especially its interactions with FuHC.

Another gene encoded in the FuHC locus, *uncle fester,* represents a partial duplication of the *fester* locus, with the genomic region encoding Exons 4–9 nearly identical to *fester's* Exons 6–11, but *uncle fester's* Exons 1–3 do not appear to be related to any *fester* sequence [35]. This protein plays a role in initiating the rejection response between incompatible individuals, but is not involved in the fusion response [35]. *Uncle fester,* like *fester,* likely acts a receptor to the FuHC ligand. Two of the *fester* exons that have statistically higher ω values than the rest of the *fester* gene (Exons 4 and 5) are not related to the *uncle fester* sequence. *Fester's* Exon 8 is very similar to *uncle fester's* Exon 6, and has higher ω values than the rest of the *fester* gene.

#### Type of selection occurring at the *fester* locus

These data do not provide unambiguous support for a specific type of selection. The polymorphism statistics are consistent with either purifying selection or directional selection. The ω statistics show that the majority of the protein is experiencing purifying selection (ω < 1), but that 15–27 codons are undergoing selection. The selection detected by ω statistics could be either balancing or directional; ω > 1 is compatible with either scenario. The distribution of variation within and among populations points towards balancing selection and away from directional selection.

The genetic basis of allorecognition has only been characterized in *B. schlosseri,* although the majority of botryllid species exhibit allorecognition. Identification and amplification of *fester* in other botryllids could allow us to discriminate between balancing and directional selection. First, we could determine if trans-species polymorphism is occurring. In several classic allorecognition systems, alleles from Species A are more closely related to alleles in Species B than they are to other alleles in Species A (*e.g.* SI loci *SRK* and *SCR* in several *Arabidopsis* species: [36,37], *Het-c* in *Neurospora crassa:*[38]). Such a pattern could be explained if alleles that pre-date speciation events have been maintained by balancing selection until the present time [39]. Second, divergence data would allow us to conduct several additional tests of selection (*e.g.* the HKA and McDonald-Kreitman tests) and apply other polymorphism statistics (*e.g.* Fu and Li's D and F).

Why might *fester* be evolving non-neutrally? If balancing selection is acting to maintain the allelic diversity at *fester,* what would be the mechanism of this selection? Given *fester's* likely function as a receptor of the ligand FuHC, *fester* may be evolving in response to FuHC evolution. Fusion can incur a significant fitness cost [40-42]; individuals with rare FuHC alleles will not fuse as often and may have higher fitness (negative frequency dependent selection). *Fester* alleles may evolve to bind with these rare FuHC alleles, and would therefore be subject to similar selective pressures as the FuHC alleles.

A recent study on the *alr2* allorecognition gene in *Hydractinia* comes to a similar conclusion [43]. They assert that *alr2* polymorphism is maintained by balancing selection, with negative frequency dependent selection as the mechanism. *Hydractinia* colonies also undergo fusion, and fusion can be costly for the losing genotype in situations where the two genotypes do not contribute equally to the next generation [43].

But if directional (rather than balancing) selection is maintaining variation at *fester*, what would be the biological explanation for this pattern? Fusion may also be beneficial to colonial ascidians such as *B. schlosseri*[41,44]*.* High rates of fusion are seen in the field (*Botrylloides violaceus*) [45] and the laboratory between unrelated individuals (*Diplosoma listerianum*) [46], and half-siblings (*B. schlosseri*) [47]. *B. schlosseri* juveniles also prefer to settle near related individuals [48,49]. In a scenario where fusion is beneficial, individuals with common *fester* alleles (and therefore higher fusion rates) would have higher fitness, and these common alleles would go to fixation.

### Relationships among *fester* haplotypes

Because B1 is paraphyletic with respect to B2, the B2 copy may be derived from a duplication of the B1 copy. In our laboratory-reared strains in which the *fester* copies have been physically mapped, both B1 and B2 reside on a single haplotype, while the A haplotype has a single copy [22]. This is consistent with a duplication event creating the second B copy. Duplication events often drive genomic diversity in vertebrate MHC receptors including *Ly49* genes in murines [50], NKG2 genes in humans [51], and lemurs [52], KIRs in humans [53,54], and heavy chain variable segment (V_H_) genes in humans [55]. However, more B2 alleles need to be sequenced before hypotheses about the origins of the B2 copy can be tested.

The A clade is equally divergent from both the B1/B2 and C clades, which are less divergent from each other than either is from the A clade. This pattern is consistent with at least 2 evolutionary scenarios: 1) the A haplotype experienced a duplication event which gave rise to the ancestor of the B1/B2 and C haplotypes, or 2) a duplication at either B1 or B2 that gave rise to the C haplotype (or vice versa). Either way, A-type alleles are nearly evenly distributed between East Coast and West Coast populations (27 vs. 24), whereas B1-type and C-type alleles are mostly found in East Coast populations (B1: 11/13, C: 17/21). This pattern suggests that the A-type alleles are more widespread in *B. schlosseri* source populations than either the B1-type or C-type alleles (both East and West Coast populations are invasive). The A-type alleles may therefore occupy the basal position in the *fester* phylogeny. But until we can sequence the *fester* locus from other *Botryllus* species*,* this remains speculation.

### Comparison of variation among *fester* allele types

The A-type alleles are more variable than either the B1-type or C-type alleles. While more A-type alleles (51) were sequenced than B1-type (13) or C-type (21) locus alleles, it is unlikely that sampling bias completely explains this pattern. We found 4 distinct A-type alleles and no such diversity was discovered in the B1, B2 or C-type alleles. There are 3 possible explanations for this pattern: 1) stronger directional or balancing selection on the A haplotype, 2) the A haplotype is older than the other haplotypes and has accumulated more diversity through neutral or selective processes and/or 3) the lack of variation in the B1/B2 haplotype may be due to homogenization of variation due to unequal crossover or gene conversion between B1 and B2 (concerted evolution) [56]. The first hypothesis is supported by the results of the polymorphism statistics, which are consistent with the action of selection on the A-type alleles but not on the B1-type or C-type alleles. We cannot evaluate the second hypothesis without sequences from other botryllid species or more *B. schlosseri* populations. Regarding the third hypothesis, concerted evolution has long been thought to play a role in the evolution of immunoglobulin genes [57,58]. For example, this process has been suggested as a mechanism for the lack of variation in certain NKG2 genes in murines and humans [59]. But concerted evolution cannot necessarily explain lower levels of variation at the *fester* C haplotype compared to the *fester* A haplotype, and the importance of concerted evolution by inter-locus gene conversion for the evolution of MHC and immunoglobulin genes has been challenged by the more recent birth-and-death model [55].

Almost all measures of A-type allele diversity are higher in the East Coast group than the West Coast group. Although we sampled a larger number of East Coast than West Coast colonies with A-type alleles (22 vs. 18), the distinct evolutionary histories of these two sets of populations may also play a role in the diversity disparity. East Coast *B. schlosseri* Lineage A came from the Mediterranean Sea, which is the center of diversity for this group of lineages collectively known as *B. schlosseri*[60]. West Coast *B. schlosseri* Lineage A, on the other hand, came from the Western Pacific Ocean [60]. The Western Pacific Ocean populations came originally from the Mediterrean Sea [60]. East Coast *B. schlosseri* may be more diverse than West Coast *B. schlosseri* because native populations seeded the East Coast and non-native populations seeded the West Coast.

## Conclusion

Despite the prevalence and importance of allorecognition systems, the genetic basis of allorecognition has rarely been characterized outside the well-known MHC in vertebrates and SI in plants. Where loci have been identified, their evolutionary history is an open question. We have identified that the *fester* locus, a putative receptor in the *B. schlosseri* allorecognition system, evolves via natural selection. Studies such as these will increase our understanding of a widespread biological phenomenon.

## Methods

### Sampling

The species *B. schlosseri* comprises five divergent lineages (A-E) [45,61]. Lineage A is thought to be native to the Mediterranean; it has spread throughout the Atlantic, Mediterranean, and Pacific Oceans through anthropogenic means. All of the *B. schlosseri* individuals in this study are Lineage A; populations from the West Coast of the U.S. originated from invasive western Pacific individuals, whereas populations from the East Coast of the U.S. originated from invasive Mediterranean individuals [60].

Colonies were collected from floating docks in each of 6 populations in 2009 and 2010: Falmouth, MA, Quissett, MA, Sandwich, MA, Monterey, CA, Santa Barbara, CA and Seattle, WA. Falmouth, MA and Quissett, MA are 3 miles apart, on Vineyard Sound and Buzzards Bay, respectively. Sandwich, MA is 25 miles from the Falmouth/Quissett area, on Cape Cod Bay. Santa Barbara, CA is 237 miles south of Monterey, CA and 1,113 miles south of Seattle, WA. Single systems were dissected from colonies and flash-frozen with liquid nitrogen and stored at −80°C.

### Amplification and sequencing

#### *Fester*

Total RNA was extracted from frozen tissue using the NucleoSpin Nucleic Acid and Protein Purification Kit (Macherey-Nagel). This RNA was used to synthesize single-stranded cDNA using SuperScript III reverse transcriptase (Invitrogen) and an oligo (dT) primer. 5-fold dilutions of the single-stranded cDNA was then PCR-amplified with TRsa and TS-PCR primers. The resulting PCR product was diluted 50-fold and used as the template for PCR amplification. We used the following primer pair to amplify *fester*: Forward: 5' AAAGATAGTGCATCTGTTTCCATCCAA 3' and Reverse: 5' GCAGCTGCTTCGATTTTCTTTCCTTGT 3'. This primer pair amplified all *fester* haplotypes, and all exons were amplified in the initial PCR. Cycling conditions were 39x (95C for 30 sec, 55C for 30 sec, 72C for 1 min 30 sec), 72C for 5 min. PCR amplification was performed in a 20-μl total reaction volume with 13.6μl of H20, 4μl of 5x HF Buffer (Finnzymes), 0.2 mM dNTPs, 0.6 μl of 100% DMSO, 0.3333 μM of each primer, 0.02U/μl of Phusion Polymerase (Finnzymes*)* and 2 μl of template DNA. PCR products were cloned using the pGEM®-T kit and at least 12 clones per colony were sequenced in order to find alleles from all allele types: many colonies have more than 1 allele type. When an A/A, B1/B2/B1/B2 or C/C homozygote was found, we religated and transformed the original PCR product and sequenced additional clones to ensure that the colony was indeed a homozygote. Colony PCR products were incubated with 0.25μl each of Exonuclease I and Shrimp Antarctic Phosphatase at 37°C for 30 min, followed by 90°C for 10 min prior to sequencing.

Purified PCR products were sequenced with a Big Dye Terminator Cycle sequencing kit and a 96 capillary 3730xl DNA Analyzer (Applied Biosystems) at the UC Berkeley Sequencing Facility. A non-redundant set of alleles has been submitted to GenBank (Accession Numbers JN083148-JN083236). Sequences were edited, trimmed and aligned with Aligner (CodonCode Corporation, Dedham, MA). Colonies sequenced for each population are shown in Table 3. Only Exons 1–5 and 8–11 were included in the alignment. We know that the PCR primers always amplified all 11 exons because the product was always 1.1 kb. However, the full-length cDNA was rarely incorporated into the bacterial vector; the longest amplicons recovered from the cloning process were almost always missing Exons 6 and/or 7 despite screening up to 192 clones per colony. Both Exons 6 and 7 were monomorphic when present, so we decided to exclude them from the alignment. No other splice variants were included in the alignment (*i.e.* all included sequences had Exons 1–5 and 8–11).

**Table 3:** ***Fester*genotypes of sequenced colonies**

**Populations**	**Number of Colonies**	**A/A**	**A/B1/B2**	**B1/B2/B1/B2**	**A/C**	**C/C**	**A/B1/B2/C**
Falmouth, MA	12	2	2	4	1	3	0
Monterey, CA	4	1	0	2	0	1	0
Quissett, MA	13	7	2	2	1	1	0
Sandwich, MA	10	1	0	1	5	2	1
Santa Barbara, CA	8	6	1	0	0	1	0
Seattle, WA	12	8	1	1	1	1	0

#### Housekeeping genes

We amplified 13 housekeeping genes (12 nuclear genes and mitochondrial cytochrome oxidase I) to determine whether the pattern of population structure and the values of polymorphism statistics were specific to the *fester* locus. Significant negative polymorphism statistics could be due to selective or demographic processes (*e.g.* recent population growth). But demographic processes would affect all genes, not just those involved in allorecognition. mtCOI is a gene commonly used for population structure analyses in *B. schlosseri* (*e.g.*[34,60,61]). Two of the 12 nuclear loci were found in GenBank (adult-type muscle actin 2, Accession #FN178504.1 and vasa, Accession #FJ890989.1) and the other 10 were located in our *B. schlosseri* EST database (40S ribosomal protein 3A, 60S ribosomal protein L6, 60S ribosomal protein L8, 60S ribosomal protein L10, 60S ribosomal protein L13, heat shock cognate 71kda protein, cytoplasmic actin 2, ADP/ATP translocase 3, heat shock protein HSP-90 beta, and vigilin).

Template for PCR amplification was generated as described above for the *fester* locus. Primers and thermocycling conditions for each gene are available from the authors. vasa PCR products were cloned as described for the *fester* locus. The PCR products of the other nuclear loci were sequenced directly. PCR products were incubated with 0.25μl each of Exonuclease I and Shrimp Antarctic Phosphatase at 37°C for 30 min, followed by 90°C for 10 min.

Purified PCR products were sequenced with a Big Dye Terminator Cycle sequencing kit and a 96 capillary 3730xl DNA Analyzer (Applied Biosystems) at the UC Berkeley Sequencing Facility. Sequences that were obtained by direct sequencing of PCR products (all nuclear sequences minus vasa) were phased in DnaSP 5.10.01 [62]. All sequences have been submitted to GenBank (40S ribosomal protein 3A: JQ596880-JQ596936, 60S ribosomal protein L6: JQ596937-JQ597084, 60S ribosomal protein L8: JQ597085-JQ597174, 60S ribosomal protein L10: JQ597175-JQ597294, 60S ribosomal protein L13: JQ597595-JQ597716, heat shock cognate 71kda protein: JQ597295-JQ597430, cytoplasmic actin 2: JQ597431-JQ597548, ADP/ATP translocase 3: JQ597549-JQ597594, heat shock protein HSP-90 beta: JQ597717-JQ597826, adult-type muscle actin 2: JQ597827-JQ597974, mtCOI: JN083237-JN083303, vasa: JN083304-JN083376, vigilin: JQ597975-JQ598070). Sequences were edited, trimmed and aligned with Aligner (CodonCode Corporation, Dedham, MA).

### Relationships among *fester* haplotypes

We used RAxML 7.2.7 on the CIPRES web portal to obtain the best-scoring ML tree, as well as bootstrap support for each node on this tree [63]. We used the GTR+G likelihood model of nucleotide substitution as implemented in RAxML. All nodes with less than 50% support were collapsed, and the resulting tree was visualized using FigTree 1.3.1 [64].

Bayesian analyses were performed with MrBayes 3.1.2 [65]. The GTR+G model of nucleotide substitution was applied (Nset=6). Each analysis was run for 10 million generations, with sampling every 1000 generations. The first 2000 trees were eliminated as burn-in. The runs were completed using the Computational Biology Service Unit at Cornell University which is partially funded by the Microsoft Corporation.

### Comparison of variation among *fester* allele types

The average number of nucleotide substitutions per site (D_xy_) and the number of net nucleotide substitutions per site (D_a_) between each pair of allele types was calculated in DnaSP 5.10.01 [62]. D_a_ corrects for within-allele-type variation [66]. The three allele types analyzed are A-type, B1-type, and C-type. For all population-level analyses, we analyze each allele type separately because each has a separate evolutionary history, and because including divergent alleles in the same data set could create artifacts. When analyzing population level data, we use the term "allele type" instead of "haplotype". B2 alleles were recovered from an insufficient number of colonies to be included in population level analyses, so our analyses were done on B1-type alleles rather than on the B1/B2 haplotype.

### Recombination

Intragenic recombination was determined in the East Coast and West Coast groups for the *fester* A-type alleles, and for the East Coast group in the *fester* B1-type and C-type alleles. Recombination was assessed by calculating R_m_, the minimum number of recombination events in DnaSP 5.10.01 [62] and the correlation between physical distance and 3 measures of linkage disequilibrium (LD): r^2^, D' and G4 in program permute [67].

### Selection inference: Distribution of polymorphism within and among populations

We characterized population structure within *B. schlosseri* for *fester* A-type, B1-type, and C-type alleles and all housekeeping genes using an analysis of molecular variance (AMOVA), fixation indices (F_ct_, F_sc_ and F_st_), and pairwise F_st_ values between populations in Arlequin 3.5.1.2 [68]. The *fester* B2-type alleles were not analyzed, as only 2 alleles were recovered from all colonies sequenced. For the *fester* A-type alleles and all the housekeeping loci, 2 groups (East Coast and West Coast) were analyzed, with 3 populations in each group (East Coast: Falmouth, MA, Quisset, MA and Sandwich, MA. West Coast: Monterey, CA, Santa Barbara, CA, Seattle, WA). Molecular variance was therefore partitioned 3 ways: among groups, among populations within groups, and within populations. For the B1-type and C-type alleles, only 1 group (East Coast) was analyzed, as few West Coast colonies had B1-type or C-type alleles. Molecular variance was therefore assigned among and within populations only.

### Tests of selection: polymorphism statistics

For East Coast and West Coast alleles separately (*fester* A-type alleles), East Coast alleles (*fester* B1-type and C-type alleles), and each of the six populations (housekeeping genes), we calculated the summary statistics θ, π, number of haplotypes, and haplotype diversity in DnaSP 5.10.01 [62]. We also employed Tajima's D [28] and Fu and Li's D* and F* [29] test statistics. Statistical significance of D, D*, and F* were determined using 10,000 coalescent simulations in DnaSP. We performed 2 sets of coalescent simulations: based on θ and segregating sites. Estimates of per gene recombination (R) for each population were made in DnaSP and were then imported into the simulations. Tajima's D, Fu and Li's D* and F* statistics were calculated for *fester* A-type alleles (East Coast and West Coast groups), B1-type alleles (East Coast group only), C-type alleles (East Coast group only), and all housekeeping genes (all six populations).

### Tests of selection: ω statistics

Omega values (ω = d_N_/d_S_) and associated 95% HPD (highest posterior density) regions across *fester* A-type, B1-type and C-type alleles were estimated using the program omegaMap 0.5 [67]. omegaMap calculates ω values in the presence of intragenic recombination [67]. omegaMap runs were carried out using the resources of the Computational Biology Service Unit at Cornell University which is partially funded by the Microsoft Corporation. We chose 250,000 iterations for each run, with thinning set to 1,000. We used an improper inverse distribution for μ, and κ, and an inverse distribution for ω and ρ. Initial parameter values for μ and κ were 0.1, and 3.0, respectively. ω and ρ priors were set between 0.01 and 100. An independent model was used for ω, so that ω values were allowed to vary across sites. The number of iterations discarded as burnin varied across runs, but was determined by plotting the traces of μ and κ; iterations affected by the starting value of the parameter were discarded. 2 independent runs were conducted for each population. These 2 runs were combined in all cases, after it was determined that the mean and 95% highest posterior density (HPD) regions for each parameter in the 2 runs matched closely.

We also calculated the posterior probability of selection per codon across the protein. Exons that contained clusters (≥ 2) of these codons were identified; Mann–Whitney U tests in R 2.12.2 were performed on these exons to determine if they had higher ω values than the rest of the protein.

## Competing interests

The authors declare that they have no competing interests.

## Authors’ contributions

MLN generated the data, performed the analyses and wrote the manuscript. AWD provided funding and edited the manuscript. Both authors have read and approved the final manuscript.

## Authors’ information

MLN studies the evolution of allorecognition in *B. schlosseri* and the evolution of reproductive isolation and speciation in the solitary ascidian *Ciona intestinalis.* AWD studies the evolution and molecular mechanisms of allorecognition in *B. schlosseri,* as well as the mechanisms of germ line parasitism in *B. schlosseri.*

## Data accessibility

All sequences have been submitted to GenBank.

## Supplementary Material

Additional file 1 **Best scoring Maximum Likelihood Tree of *fester *A, B1, B2 and C allele types.** Blue = A-type alleles, Orange = B1-type alleles, Purple = B2-type alleles, Black = C-type alleles. FLM = Falmouth MA, MR = Monterey CA, QST = Quissett MA, SW = Sandwich MA, SB = Santa Barbara CA, SE = Seattle WA. Numbers are bootstrap values from 1,000 replicates. Click here for file

Additional file 2 AMOVA, Fixation Indices and Pairwise Fst values for fester and all housekeeping genes. Click here for file

Additional file 3 Tajima's D, Fu and Li's D* and F* statistics for all Housekeeping Genes. Click here for file
