# Supplementary figures and images for: The fester locus in Botryllus schlosseri experiences selection
Source: BMC Evol Biol. 2012 Dec 22;12:249. doi: 10.1186/1471-2148-12-249 (PMC3549757; doi:10.1186/1471-2148-12-249)

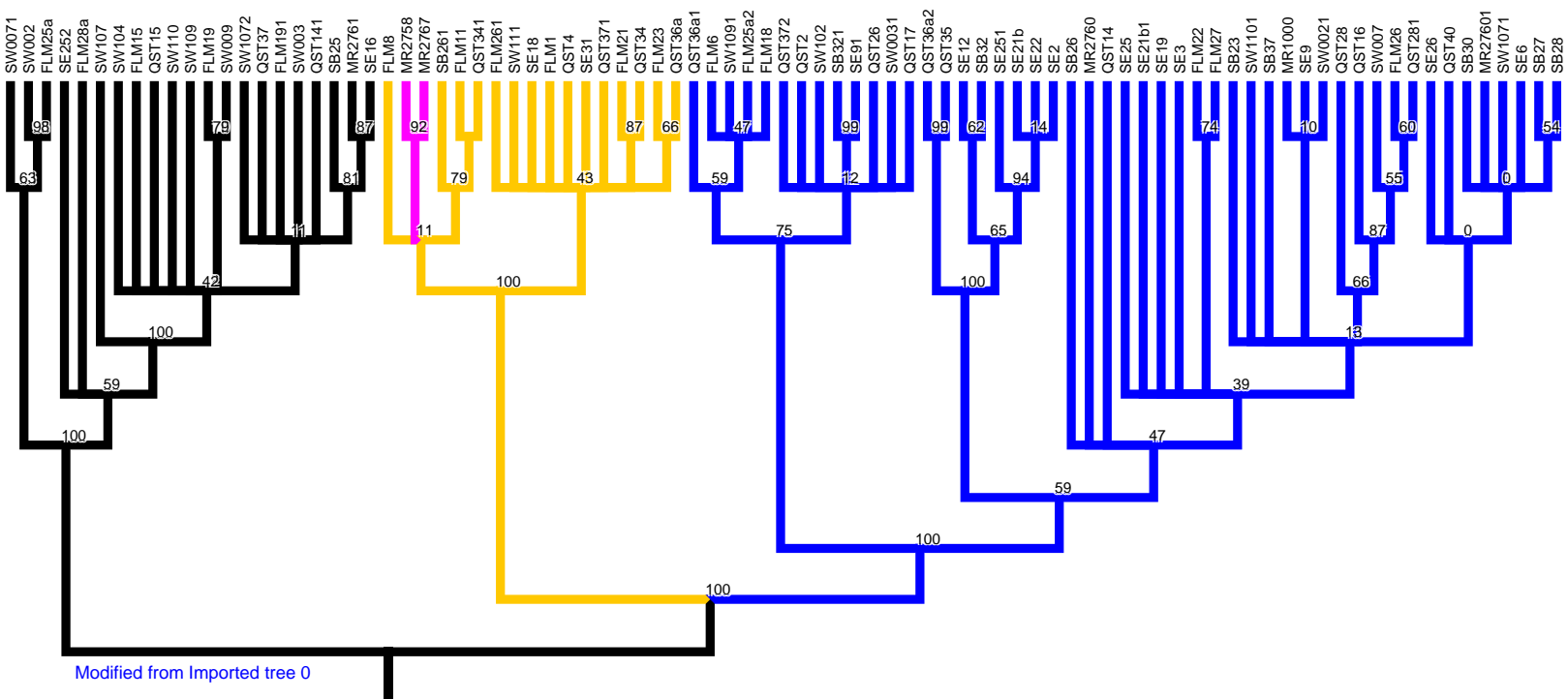

Supplement: Additional file 1 — Best scoring Maximum Likelihood Tree of fester A, B1, B2 and C allele types. Blue = A-type alleles, Orange = B1-type alleles, Purple = B2-type alleles, Black = C-type alleles. FLM = Falmouth MA, MR = Monterey CA, QST = Quissett MA, SW = Sandwich MA, SB = Santa Barbara CA, SE = Seattle WA. Numbers are bootstrap values from 1,000 replicates. [file 1471-2148-12-249-S1.pdf]
